# Limitations of cardiac risk scores in elective non-cardiac surgeries: a Brazilian cohort

**DOI:** 10.1590/1806-9282.20251821

**Published:** 2026-06-26

**Authors:** Fabio Figueiredo Costa, Liz Lustoza Brandão, Fernanda Hora Gomes de Sá, Paulo Átila Castro Carvalho de Jesus, João Vitor Souza Borges, Anna Claudia Monteiro Luz Santos, Jennifer do Carmo Souza Pinheiro, Lívia Brito Oliveira, Glicia Gleide Gonçalves Gama, Adriana Lopes Latado

**Affiliations:** 1 Universidade Federal da Bahia, Hospital Universitário Professor Edgard Santos, Empresa Brasileira de Serviços Hospitalares, Cardiorespiratory System Unit – Salvador (BA), Brazil.; 2 Secretaria da Saúde do Estado da Bahia – Salvador (BA), Brazil.; 3 Universidade Estadual de Campinas – Salvador (BA), Brazil.; 4 Universidade Federal da Bahia – Salvador (BA), Brazil.; 5 Foundation José Silveira – Salvador (BA), Brazil.; 6 Universidade Federal da Bahia, Hospital Universitário Professor Edgard Santos, Empresa Brasileira de Serviços Hospitalares, Research and Technological Innovation Management Sector – Salvador (BA), Brazil.; 7 Universidade Federal da Bahia, Hospital Universitário Professor Edgard Santos, Empresa Brasileira de Serviços Hospitalares, Medical Clinic Unit – Salvador (BA), Brazil.; 8 Universidade Federal da Bahia, Hospital Universitário Professor Edgard Santos, Empresa Brasileira de Serviços Hospitalares, Faculdade de Medicina, Cardiorespiratory System Unit and Research and Technological Innovation Management Sector – Salvador (BA), Brazil.

**Keywords:** Risk assessment, General surgery, Cardiovascular diseases

## Abstract

**INTRODUCTION:**

Surgical procedures are associated with an excess of postoperative cardiovascular events. Risk scores are used for cardiovascular stratification but lack proper validation across diverse populations.

**OBJECTIVE:**

The aim of this study was to assess the accuracy of the Revised Cardiac Risk Index, American College of Physicians score, and intuitive clinical judgment in predicting postoperative cardiovascular complications.

**METHODS:**

In this prospective, longitudinal study, adults assessed for cardiovascular risk undergoing elective non-cardiac surgery were included. Perioperative clinical variables, Revised Cardiac Risk Index, American College of Physicians, and clinical judgment were evaluated, as well as in-hospital and 30-day postoperative outcomes. Data were analyzed using descriptive statistics, and score performance was assessed by C-statistics.

**RESULTS:**

A total of 420 participants, with a mean age of 64.1±12.6 years, were studied, of which 47.1% were female and 71.2% were Black/Hispanic. Among the studied patients, 74.3% had hypertension, 32.9% were diabetics, 13.1% had heart failure, 11.2% had coronary artery disease, and 7.9% had stroke. Preoperative risk levels (low/medium/high) were as follows: Revised Cardiac Risk Index 88%/10.5%/1.4%; American College of Physicians 71.2%/27.4%/1.4%; and clinical judgment 64.5%/33.1%/2.4%. During hospitalization, there were 11 deaths, 3 myocardial infarctions, and 1 acute pulmonary edema. The risk scores showed poor discrimination for death and combined in-hospital cardiovascular outcomes, with area under the receiver operating characteristic curve <0.70. Cardiologist judgment performed slightly better than the scores. Results were similar for major and non-major surgeries, and when evaluating only patients aged 50 years or older.

**CONCLUSION:**

Cardiac risk assessment demonstrated low accuracy in predicting in-hospital postoperative cardiovascular outcomes, regardless of surgical magnitude.

## INTRODUCTION

Perioperative cardiac complications in non-cardiac surgeries account for a considerable absolute number of adverse events, given the high number of procedures performed annually in Brazil^1^. Risk stratification scores are applied in clinical practice to estimate the likelihood of major cardiovascular complications, cardiovascular death, and all-cause mortality^2,3,4,5^. These tools enhance patient care by providing evidence-based information to support clinical decision-making and mitigate adverse outcomes^1^.

Subjective clinical judgment remains valuable in assessing perioperative cardiovascular risk; however, validated risk scores facilitate a more rational application of complementary tests and guide the requirement for cardiology support^1^.

In Brazil, the surgical population is becoming older, while minimally invasive procedures with lower intrinsic risk have been increasingly adopted. Endoscopic procedures and low-complexity surgeries, such as cataract extraction, are also rising, but these scenarios are not assessed by traditional risk scores^1,2,3,4^.

There are few scientific publications regarding the accuracy and validation of cardiac surgical risk assessment in non-cardiac procedures in low- and middle-income countries. Fatal and non-fatal postoperative complications, both hospital-acquired and long-term, can be elevated in this setting, especially for elderly patients^6,7^. In Brazil, the surgical population is aging, while minimally invasive procedures with lower intrinsic risk are increasingly being adopted. Endoscopic procedures and low-complexity surgeries, such as cataract extraction, are also on the rise, but these scenarios are not assessed using traditional risk scores^1,2,3,4^.

This study aimed to compare the performance of two risk stratification tools commonly applied in Brazil—the Revised Cardiac Risk Index (RCRI) and the American College of Physicians (ACP) index^1^—with clinical judgment for predicting perioperative cardiovascular complications in patients undergoing non-cardiac surgery at a university hospital in Northeastern Brazil, stratified by surgical complexity.

## METHODS

### Design, sample, and eligibility

This study was conducted at a federal university hospital in Bahia, Brazil, a referral center for medium- and high-complexity care serving the entire state of Bahia. It was a prospective, observational, analytical cohort study including patients undergoing non-cardiac surgery at the institution.

Participants were recruited through a convenience sampling method. The sample comprised outpatients evaluated in a clinic dedicated to preoperative cardiac risk assessment and inpatients referred for preoperative cardiological evaluation between April 2019 and December 2023. All clinical assessments were performed by three experienced cardiologists, members of the research team and regular staff of the hospital.

Eligibility criteria involved patients aged ≥18 years, referred for cardiovascular risk assessment, and undergoing elective non-cardiac surgery, performed at the study hospital. Major surgeries included suprainguinal vascular, intra-abdominal, intrathoracic, and neurosurgical procedures.

Patients were excluded if they did not undergo surgery for any reason or if the surgery was performed in other health facilities. Cases of unobtainable 30-day postoperative outcome data (e.g., incorrect or incomplete contact information) were assessed for in-hospital outcomes only.

### Study procedures

An interview was conducted for all participants. Data were collected through structured case report forms, performed during patient interviews and supplemented by reviewing medical records. Variables included demographic and clinical characteristics, current medications, laboratory and imaging findings, cardiac risk assessment by RCRI and ACP scores, subjective clinical judgment of the cardiologist, and clinical outcomes.

Postoperative outcomes were recorded during hospitalization and reassessed 30 days after surgery by telephone contact. When phone contact was unsuccessful, information was obtained from checking physicians or hospital records.

*Cardiac risk scores*

Both scores, ACP and RCRI scores, were calculated as recommended by the Perioperative Cardiovascular Assessment Guideline of the Brazilian Society of Cardiology, 2024^1^. The ACP index categorizes patients into low (<3%), intermediate (3–15%), or high (>15%) risk for postoperative coronary events^4,8^, while RCRI was stratified into classes I (0.4% risk), II (0.9% risk), III (6.6% risk), and IV (11% risk)^2,8^. Major cardiac complications considered in the RCRI include myocardial infarction (MI), pulmonary edema, ventricular fibrillation or primary cardiac arrest, and complete heart block within 5 postoperative days^2,8^.

### Outcomes of interest

Study outcomes were aligned with the RCRI and ACP scores: fatal and non-fatal acute MI, acute pulmonary edema, ventricular fibrillation or primary cardiac arrest, and complete heart block. Outcomes were assessed up to postoperative day 5, with additional events recorded up to 30 days after surgery. The research team that evaluated outcomes was different from the professionals who performed the preoperative cardiac risk assessment, although formal data blinding strategies between the teams were not applied.

### Statistical analysis

Quantitative variables were summarized using mean±standard deviation, or median±interquartile range, while categorical variables were described using proportion. Normality of continuous data was assessed using the Shapiro-Wilk test and distributional characteristics (kurtosis and skewness). Discriminatory ability of the scores for predicting cardiovascular outcomes was evaluated using the C-statistic (area under the ROC curve), comparing the RCRI, ACP, and clinical judgment. Calibration was tested with the Hosmer-Lemeshow method. Surgical procedures were classified as major or non-major for subgroup analyses. Statistical analyses were performed using R software (R Core Team, Vienna, Austria), Windows version (open-source).

### Ethical considerations

The study protocol was approved by the institutional Research Ethics Committee (approval no. 3.186.630; CAAE no. 05853319.2.0000.0049; March 8, 2019). The study complied with Brazilian National Health Council Resolution 466/2012 and its operational guidelines. Written informed consent was obtained from all participants.

## RESULTS

A total of 420 patients undergoing elective non-cardiac surgery between April 2019 and December 2023 were included. Their mean age was 64.1±12.6 years; 47.1% were female and 71.2% self-identified as Black or mixed-race. Regarding comorbidities, 74.3% had hypertension, 32.9% diabetes mellitus, 45.7% dyslipidemia, and 13.1% prior heart failure. Additionally, 11.2% had a history of coronary artery disease and 7.9% had a previous stroke. Baseline demographic, clinical, and laboratory characteristics are shown in Table 1.

**Table 1:** Baseline clinical, demographic, and laboratory characteristics of the sample.

Characteristic	N=420
Age		
Mean (SD)	64.1 (12.6)
Median (IQR)	65.5 (57–62)
Female sex, n (%)	198 (47.1)
Race/ethnicity, n (%)		
Black	151 (36.0)
Brown (mixed)	148 (35.2)
White	120 (28.6)
Comorbidities, n (%)					
Hypertension	312 (73.4)
Diabetes mellitus	138 (32.9)
Dyslipidemia	192 (45.7)
Coronary artery disease	47 (11.2)
Heart failure	54 (13.1)
Stroke/TIA	33 (7.9)
Hemodynamic data, mean (SD)			
Heart rate	77 (12.7)
Systolic blood pressure	138 (20.3)
Diastolic blood pressure	81.4 (11.3)
BMI, mean (SD)	27.5 (5.92)
Laboratory tests			
Urea, mg/dLa	40.4 (25.4)
Creatinine, mg/dL^a^	1.3 (1.4)
Sodium, mEq/L^a^	139 (3.7)
Potassium, mEq/L^a^	4.5 (0.6)
Glucose, mg/dL			
Mean (SD)	112 (45.5)
Median (IQR)	100 (88–120)
Hemoglobin, g/dL^a^	13.1 (2.0)
Hematocrit, %^a^	39.2 (5.8)

SD: standard deviation; IQR: interquartile range; TIA: transient ischemic attack; BMI: body mass index; SBP: systolic blood pressure; DBP: diastolic blood pressure.

^a^Mean (SD).

Concerning surgical procedures, 17.4% were classified as major surgeries. General abdominal surgery represented 22.3% of the cases, of which 7.8% were major. Besides, only 1.2% of the patients underwent intrathoracic procedures and 2.2% underwent neurosurgery. Suprainguinal vascular procedures were performed in 6.2% and infrainguinal vascular procedures in 10.3% of the patients. Carotid procedures accounted for 0.7%, intracerebral arterial surgeries for 1.9%, and arteriovenous fistula creation for 2.6%. Low-risk procedures such as endoscopies and ophthalmologic or dermatologic surgeries represented 33.4% within the sample.

### Preoperative risk prediction by clinical scores

The predictive performance of both RCRI and ACP algorithms was evaluated for in-hospital and 30-day postoperative cardiovascular outcomes. Based on available data from 418 patients, risk stratification using RCRI showed that 368 patients (88.0%) were at low risk, 44 patients (10.5%) were at intermediate risk, and 1.4% were at high risk. In the same way, the ACP score (n=420) classified the majority of patients as low risk (71.2%), followed by 27.4% as intermediate risk and then 1.4% as high risk. Compared with the RCRI, subjective clinical judgment by cardiologists yielded higher proportions of patients categorized as intermediate risk (33.1%), with fewer classified as low risk (64.5%).

Stratification by surgical complexity showed that high-risk classification was observed at a higher rate among major surgeries versus non-major surgeries according to the RCRI (5 vs. 0.8%) and clinical judgment (4.7 vs. 2%), but not with the ACP algorithm, which showed similar distributions between major and non-major surgeries (1.6 vs. 1.4%, respectively).

### In-hospital and 30-day cardiovascular outcomes

Clinical outcomes included death, non-fatal, and fatal MI, pulmonary edema, complete atrioventricular block, ventricular fibrillation, ventricular tachycardia, and heart failure. During hospitalization, there were 11 deaths (2.6%), 3 cases of non-fatal MI (0.7%), and 2 cases of pulmonary edema (0.7%), one of which occurred concomitantly with MI. One additional new-onset case of heart failure was observed after discharge within 30 days, with no further adverse events during this period.

Among the 15 in-hospital events, 6 occurred in patients undergoing major surgery and 9 in those undergoing non-major surgery. According to the RCRI, adverse events occurred in 2.7% of low-risk and 11.4% of intermediate-risk patients. For the ACP score, corresponding rates were 3.3 and 4.3%, respectively. No events were recorded among patients classified as high risk by either score. Clinical judgment classified low-risk patients with 1.8% event rates and intermediate-risk patients with 7.2%, while no events were observed among those deemed high risk.

In major surgeries (n=64), six in-hospital cardiovascular events were observed. Event rates among patients classified as low risk were 8.3% (RCRI), 8.7% (ACP), and 6.7% (clinical judgment). Among intermediate-risk patients, event rates were 15.4% (RCRI), 11.8% (ACP), and 12.9% (clinical judgment).

In non-major procedures, event rates among low-risk patients were 1.9% (RCRI), 2.4% (ACP), and 1.2% (clinical judgment). For intermediate-risk patients, rates were 9.7, 3.1, and 5.6%, respectively.

### Accuracy of risk scores in predicting mortality and postoperative cardiovascular outcomes

For the RCRI, combined endpoints included death, heart failure, ventricular arrhythmia (VF/VT), pulmonary edema, and MI. For the ACP score, endpoints were death and non-fatal MI. As only one cardiovascular event occurred after hospital discharge, 30-day outcomes were not included in discrimination analyses.

**Figure 1:**
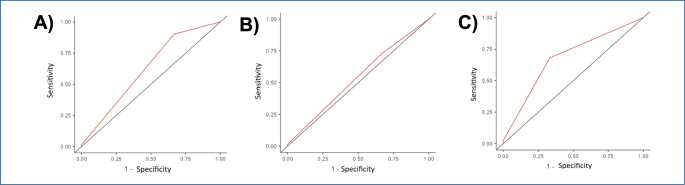
Areas under the receiver operating characteristic curve for the association between risk score levels and postoperative inhospital clinical outcomes in patients undergoing elective non-cardiac surgery. (A) Revised Cardiac Risk Index score=0.62; (B) American College of Physicians score=0.54; (C) clinical judgment=0.68.

As shown in Figure 1, both RCRI and ACP demonstrated poor discriminatory ability (area under the curve [AUC]: 0.62 and 0.54, respectively), with the ACP score performing worse. Cardiologists’ clinical judgment showed slightly better performance, though still limited, with an AUC of 0.68 for postoperative cardiovascular events (Figure 1).

**Figure 2:**
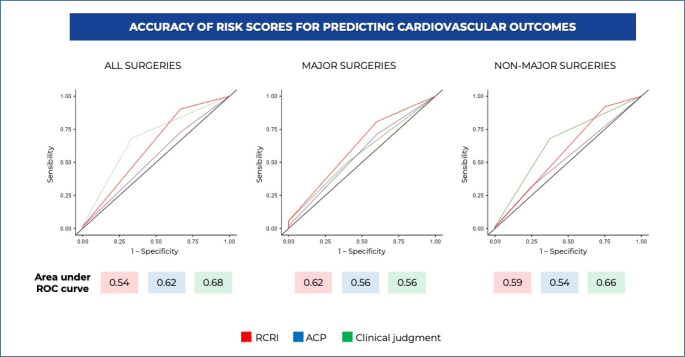
Comparison of the performance of Revised Cardiac Risk Index and American College of Physicians scores, and the cardiologist’s clinical judgment, in the total sample, in major surgery and in non-major surgery.

Additional ROC analyses stratified by surgical complexity (major vs. non-major) yielded similar findings (Figure 2). Sensitivity analyses restricted to patients aged ≥50 years— closer to the populations used in original score validations— also demonstrated poor discrimination across all tools, with AUCs ranging from 0.51 to 0.66.

## DISCUSSION

This study evaluated a sample of patients undergoing non-cardiac surgery, aiming to assess the accuracy of two widely used cardiac risk scores in both national and international clinical practice, and the risk assessment provided by experienced cardiologists. The results demonstrated that both the RCRI and ACP scores had low prediction for fatal and non-fatal in-hospital cardiovascular events, with areas under the ROC curve <0.70. The incidence of in-hospital outcomes was 4%, with 2.6% mortality, and only one case of new-onset heart failure occurred after hospital discharge within 30 days post-surgery. These rates were lower than those reported by Heinisch et al., who observed cardiovascular complications in 16% of their sample, with no cardiovascular deaths reported^8^. This difference may be explained by the type of surgeries performed (more than 60% major procedures in Heinisch’s results, compared with 17.4% in ours), baseline population characteristics, comorbidities, treatment patterns (including greater statin use in our cohort), less invasive surgical techniques, and safer contemporary anesthetic protocols.

In our study, most patients were classified as low risk by preoperative risk scores, yet cardiologists’ subjective assessment led to a significant increase in intermediate-risk classification, particularly when compared with the RCRI score. Surgical magnitude, especially major procedures, appeared to influence the perceived risk level by subjective analysis. Clinical judgment, although susceptible to both intuitive and analytical biases, showed slightly higher accuracy than the risk scores, albeit with unsatisfactory performance (ROC curve=0.68). Devereaux et al. similarly highlighted these discrepancies, reporting weak agreement (kappa=0.38) between physicians’ subjective estimates and validated risk scores for non-cardiac surgery^9^. In their study, physicians overestimated risk in 16% of patients—likely generating unnecessary costs from additional diagnostic testing—and underestimated risk in 13%, potentially leading to insufficient perioperative evaluation and increased adverse outcomes. Although probabilistic risk models are not perfect, they are superior to the subjective judgments of physicians because human judgment is based on experience and intuition and is therefore susceptible to cognitive biases. These mental shortcuts, while useful in daily decision-making, can lead to distortions and errors in perioperative risk assessment^10^.

The RCRI was originally derived from a prospective single-center cohort of 2,893 patients aged ≥50 years undergoing elective major non-cardiac surgery, with no inclusion of low-complexity procedures—differing substantially from our sample^2^. The RCRI showed moderate accuracy in its original validation cohort (ROC area=0.812)^2^. However, subsequent studies applying the RCRI in heterogeneous populations reported lower predictive values, with ROC area varying between 0.62 and 0.69^11^. These values, much lower than those in the original cohort, align more closely with our findings. Several factors may explain this difference. First, our study included many patients undergoing low-risk procedures, in contrast to the original RCRI cohort. Second, we included patients aged ≥18 years, whereas the RCRI was derived only from patients ≥50 years. In this regard, however, even in sub-analyses of patients over 50 years of age, the RCRI score did not prove to be accurate. Finally, advances over the past 25 years—including widespread laparoscopic and endovascular techniques and improved anesthesia—differ significantly from the context of the original RCRI derivation cohort, which primarily underwent traditional open surgeries.

Heinisch et al. also reported poor performance of preoperative scores—ASA, Detsky, Goldman, and Larsen—with ROC areas of 0.48, 0.38, 0.48, and 0.49, respectively, inferring that cardiac risk indices have limited value for predicting perioperative cardiac events, despite their widespread clinical use^12^. Applying these scores to populations markedly different from their derivation cohorts may partly explain these results. Additionally, the lack of systematic postoperative cardiovascular monitoring—such as serial electrocardiograms and biomarkers (troponin or creatine kinase [CK]-MB)—as well as changes in the definition of acute MI over time, may also play a significant role in differences between study results.

Cardiac complications in the perioperative setting of non-cardiac surgery remain a major concern. The use of traditional risk scores—such as RCRI and modified ACP—or cardiologists’ subjective assessments demonstrates limited accuracy in populations dominated by low-risk procedures. Careful interpretation of these results is required to avert unnecessary use of medical resources and the unwarranted postponement or cancellation of essential surgical procedures. The recently proposed Beirut score offers ease of application and requires minimal diagnostic resources, with greater representation of low-risk procedures in its derivation cohort. However, like other perioperative scores, it originated from a single US center, which raises concerns about external validity and applicability in other settings, including Brazil^5^.

In current clinical practice, patients deemed “low risk” by established scores may still face a significant risk of adverse consequences not captured by these models^2,5,13,14^. Several comorbidities identified during preoperative assessment—such as obesity, smoking, or chronic lung disease—are not included in existing scores, despite their potential impact on both short- and longterm perioperative outcomes^14,15^.

New algorithms are currently being tested for predicting adverse outcomes in different clinical and surgical scenarios, some with the addition of machine learning technology, which have shown promise^16,17^. The incorporation of new analytical models, sometimes in addition to existing predictive tools, is important to update data on cohorts from different clinical and surgical contexts and to encompass more complex innovative technologies.

This study has limitations. First, the research took place in a single center, which may limit the generalizability of the findings. The lack of complete blinding of the evaluators in assessing hospital outcomes has the potential for bias, even though the verification of postoperative events was carried out by a research team different from the cardiologists who performed the preoperative risk assessment. The inferential statistical analyses were exploratory because a priori sample size was not calculated. On the other hand, our findings provide real-world clinical data from a highly heterogeneous population. Understanding this reality supports the implementation of strategies to improve perioperative care, optimize resource utilization, and reduce unnecessary surgical cancellations or delays. These improvements may ultimately have a positive economic impact, particularly in low- and middle-income countries such as Brazil.

In conclusion, this study found that preoperative cardiac risk assessment for predominantly low-complexity non-cardiac surgeries, whether based on ACP, RCRI, or expert clinical judgment, showed poor accuracy in predicting major fatal and non-fatal postoperative cardiovascular events, particularly in-hospital outcomes. The development of new risk scores, likely by incorporating machine learning insights, is warranted to improve patient stratification, minimize unnecessary testing, and avoid inappropriate postponement or cancellation of essential surgical procedures.

## Data Availability

The datasets generated and/or analyzed during the current study are available from the corresponding author upon reasonable request.
